# Association of Adipokines with Insulin Resistance, Microvascular Dysfunction, and Endothelial Dysfunction in Healthy Young Adults

**DOI:** 10.1155/2015/594039

**Published:** 2015-10-13

**Authors:** Cynthia Cheng, Constantine Daskalakis

**Affiliations:** ^1^Department of Family and Community Medicine, Thomas Jefferson University, Philadelphia, PA 19107, USA; ^2^Department of Pharmacology and Experimental Therapeutics, Division of Biostatistics, Thomas Jefferson University, Philadelphia, PA 19107, USA

## Abstract

Proinflammatory adipokines (inflammation markers) from visceral adipose tissue may initiate the development of insulin resistance (IR) and endothelial dysfunction (ED). This study's objective was to investigate the association of five inflammation markers (CRP and four adipokines: IL-6, TNF*α*, PAI-1, and adiponectin) with IR (quantitative insulin resistance check index (QUICKI)), microvascular measures (capillary density and albumin-to-creatinine ratio (ACR)), and endothelial measures (forearm blood flow (FBF) increases from resting baseline to maximal vasodilation). Analyses were conducted via multiple linear regression. The 295 study participants were between 18 and 45 years of age, without diabetes or hypertension. They included 24% African Americans and 21% Asians with average body mass index of 25.4 kg/m^2^. All five inflammation markers were significantly associated with QUICKI. All but adiponectin were significantly associated with capillary density, but none were associated with ACR. Finally, IL-6 and PAI-1 were significantly associated with FBF increase. We also identified a potential interaction between obesity and IL-6 among normal-weight and overweight participants: IL-6 appeared to be positively associated with QUICKI and capillary density (beneficial effect), but the inverse was true among obese individuals. These study findings suggest that inflammation measures may be potential early markers of cardiovascular risk in young asymptomatic individuals.

## 1. Introduction

A large body of scientific evidence supports the critical role of inflammation markers (including adipokines, bioactive mediators secreted directly from adipocytes, and vascular cells within adipose tissue) in the development of systemic inflammation that contributes to vasculopathy and cardiovascular risk within obese individuals [1, 2]. More specifically, proinflammatory adipokines from visceral adipose tissue may initiate the development of insulin resistance, microvascular dysfunction, and endothelial dysfunction. Insulin resistance and endothelial dysfunction have both been implicated in the pathogenesis of hypertension [3–7] and atherosclerosis [8, 9]. Microvascular dysfunction may affect both peripheral vascular resistance and insulin-mediated glucose disposal, thereby contributing to hypertension and insulin resistance, respectively [10].

The majority of studies examining the association of adipokines with factors related to increased cardiovascular risk have been conducted in older and obese subjects [11]. However, identification of clinically useful predictors for detecting younger, asymptomatic individuals at risk for developing microvascular and macrovascular disease might provide critical opportunities for early intervention and prevention. Accordingly, the objective of this study was to investigate whether or not evidence of early pathogenesis involving inflammation markers is present in healthy young adult individuals, without diabetes or hypertension. More specifically, we assessed whether adipokines are associated with insulin sensitivity/resistance measured with the quantitative insulin sensitivity check index (QUICKI); microvascular baseline (resting) and maximal capillary density; renal and generalized microvascular function, measured with the albumin-to-creatinine ratio (ACR); and endothelial dysfunction measured with the increase in forearm blood flow from resting baseline to maximal vasodilation (FBF % increase).

## 2. Materials and Methods

### 2.1. Study Design and Population

The study was designed to evaluate the role of inflammation markers in the development of insulin resistance, microvascular dysfunction, and endothelial dysfunction and eventually the progression to (pre)diabetes and (pre)hypertension. The study protocol was approved by the Thomas Jefferson University Institutional Review Board and written informed consent was obtained from all study subjects. Participants were recruited between July 2011 and February 2014, in Philadelphia, Pennsylvania, from (i) the patient population of a large urban academic family medicine outpatient practice serving 40,000 individuals and (ii) a cohort of 500 young adult African Americans enrolled in prior investigations of cardiovascular risk. The main eligibility criteria were (i) age between 18 and 45; (ii) no clinically diagnosed hypertension, that is, systolic blood pressure (SBP) < 140 mm Hg and diastolic blood pressure (DBP) < 90 mm Hg; (iii) no clinically diagnosed diabetes. All study participants have completed a baseline assessment and are now undergoing a follow-up assessment 2-3 years after enrollment. In this paper, we used data collected during the baseline assessments to evaluate the association of inflammation markers with insulin sensitivity/resistance and with microvascular and endothelial outcomes.

### 2.2. Data Collection and Study Measures

Information was collected on participants' sociodemographics, family history of diabetes and cardiovascular disease, and anthropometric measures. Body mass index (BMI) was computed as weight divided by height squared (kg/m^2^). Finally, both serum and urine samples were obtained and analyzed. Blood pressure was also measured, although it is not the focus of this paper.

### 2.3. Adipokine (Adiponectin, IL-6, PAI-1, and TNF*α*) and CRP Measurement

The adipokine and CRP assays were performed using commercially available ELISA kits. IL-6 was measured with a kit from R&D Systems (Minneapolis, MN), with intra-assay coefficient of variation (CV) 4% and interassay CV 6%. PAI-1 was measured with the Elitest kit from DiaPharma (West Chester, OH), with intra-assay CV 5% and interassay CV 5%. Kits from R&D Systems were also used for assays for TNF*α* (intra- and interassay CVs both 5%) and adiponectin (intra- and interassay CVs both < 10%). Finally, hsCRP was also measured with a kit from R&D Systems (intra- and interassay CVs = 5% and 6%, resp.).

### 2.4. Glucose and Insulin Measurement

Glucose was analyzed by the glucose oxidase technique with the Glucostat analyzer (YSI, Model 27), which was calibrated routinely. Insulin was assayed using a solid phase radioimmunoassay (“Coat-A-Count” from Diagnostic Products Corporation, Los Angeles, CA). The quantitative insulin sensitivity check index (QUICKI) of insulin sensitivity was computed as the reciprocal of the sum of the log fasting glucose (mg/dL) and the log fasting insulin (*μ*U/mL): QUICKI = 1/{log fasting glucose (mg/dL) + log fasting insulin (*μ*U/mL)} [12]. Higher QUICKI values indicate greater insulin sensitivity.

### 2.5. Microvascular Measures (Capillary Density)

The capillaroscopy technique was adapted from Serné et al. [13], and details of our protocol have been previously published [14–16]. In brief, skin nailfold capillaries in the dorsal third finger were visualized at 38.4x magnification (Olympus stereo microscope SZX16, Center Valley, PA), linked to a Retiga 2000R monochrome digital camera (QImaging; Surrey, BC) and a PC computer. The nailbed was illuminated with a 250 W halogen fiber optic lamp (KL 2500 LCD: Schott-Fostec; Elmsford, NY), with additional illumination from a 150 W fiberoptic halogen light (B&B Microscopes, Ltd.; Warrendale, PA) in darkly pigmented individuals. We photographed (i)* baseline (resting) capillary density*, which represents continuously perfused capillaries [13], and (ii)* venous occlusion (maximal capillary density)*, which represents maximal visualization of all capillaries present, including both perfused (with active red blood cell motion) and nonperfused (filled with stagnant, nonmoving red blood cells) capillaries [17]. We utilized a computer-based method for quantifying capillary density using Image-Pro Premier imaging software (Version 9.1, Media Cybernetics, Inc., Bethesda, MD) [14, 15]. Higher capillary densities indicate better microvascular structure. We have found capillaroscopy measurements performed by trained personnel to be highly reliable, with intraclass correlation coefficients generally exceeding 0.9 [18].

### 2.6. Urinary Albumin Excretion

In addition to capillaroscopy, we also assessed renal and generalized microvascular function through urinary albumin excretion [19], specifically the albumin-to-creatinine ratio (ACR). Urinary albumin excretion was determined using a radioimmunoassay (Diagnostic Products Corporation, Los Angeles, CA) on timed overnight (approximately 8 hours) urine collections. Albumin greater than 20 *μ*g/min and ACR greater than 30 *μ*g albumin per mg creatinine are considered indicative of microalbuminuria.

### 2.7. Endothelial Function

We followed a well standardized, noninvasive method of postischemic flow mediated vasodilation [20]. This approach assesses the entire forearm vasculature rather than a single large artery. Forearm blood flow (FBF) was measured at (i)* rested baseline* (FBF_base_) and (ii)* hyperemic induced maximal vasodilation *(FBF_max_). FBF_max_ and the ratio of FBF_max_/FBF_base_ are accepted noninvasive measures of endothelial function [21]. We analyzed a variation of the FBF ratio, the percent increase between the FBF_base_ and FBF_max_, computed as (FBF_max_ − FBF_base_)/  FBF_base_ × 100. Greater increases in FBF indicate better endothelial function.

### 2.8. Data Analyses

The objective of these analyses was to assess the association of inflammation markers (CRP, IL-6, TNF*α*, PAI-1, and adiponectin) with

- insulin sensitivity/resistance, measured with the quantitative insulin sensitivity check index (QUICKI);
- microvascular structure, as reflected in the capillary density at rest (baseline capillary density) and after venous occlusion (maximal capillary density);
- renal and generalized microvascular function, as reflected in the albumin-to-creatinine ratio (ACR);
- endothelial function, measured with the increase in forearm blood flow from resting baseline to maximal vasodilation (FBF % increase).

We hypothesized that higher levels of CRP, IL-6, TNF*α*, and PAI-1 would have worse profiles with respect to these outcomes (i.e., low QUICKI, low capillary densities, high ACR, and small FBF increase), while the reverse would be true for adiponectin.

We used multiple linear regression to analyze each outcome separately. All outcomes except for ACR were analyzed on the original scale, but ACR was analyzed after log-transformation because of its skewed distribution. For each outcome, the main model included all five inflammation measures as predictors (since we aimed to estimate the independent effect of each marker), as well as age, sex, race, marital status, employment status, smoking, drinking, and BMI (classified as normal, overweight, or obese). The models for the microvascular and endothelial outcomes also controlled for QUICKI. In additional exploratory analyses, we evaluated the potential interaction between BMI and inflammation markers with respect to insulin resistance and microvascular outcomes. The data analyses were conducted in SAS 9.4 and Stata 13.1.

## 3. Results and Discussion

### 3.1. Results

The study enrolled a total of 312 subjects. After exclusion of 17 subjects (2 who were found to be ineligible after enrollment and 15 because of missing data on inflammation measures and/or microvascular and endothelial outcomes), these analyses were based on 295 subjects.

Table 1 summarizes the characteristics of the study participants who were relatively young (18 to 45 years old), primarily women, and included sizable numbers of both African Americans and Asian Americans. Most participants were normotensive, with only about one-fifth having blood pressure in the prehypertensive range.

Table 2 summarizes the inflammation measures (CRP and four adipokines), as well as the study outcomes. In addition, it also summarizes a number of related measures to more fully characterize the participants, for example, glucose as a measure of diabetes status, the homeostatic model assessment index (HOMA) as another measure of insulin resistance, and forearm vascular resistance as another measure of endothelial function. Most participants were nondiabetic, although blood glucose appeared to be in the prediabetic range for about a quarter of them.

Average capillary density was about 37 capillaries per mm^2^ at rest and 55 capillaries per mm^2^ after venous occlusion. Mean ACR was 2.7 mg/g, with only 2 individuals having values consistent with microalbuminuria, based on either albumin excretion or ACR (one individual had albumin 1 *μ*g/min but ACR 33.5 mg albumin per g creatinine, and another individual had albumin 23 but ACR 13.3). There was almost a 9-fold average increase in FBF from baseline to maximal vasodilation (mean change = 795%).

Table 3 presents the univariable associations (correlation coefficients) between the five inflammation measures and the five study outcomes. However, these results may be confounded by a number of other participant characteristics, hence the need to adjust for them in multivariable modeling.

### 3.2. Insulin Sensitivity/Resistance (QUICKI)

Table 4 summarizes the results of the multivariable analysis relating the five inflammation measures to insulin sensitivity/resistance. The results are expressed as the average mean difference in QUICKI associated with a difference of about 1 standard deviation in each marker's levels. For example, for each additional 8 *μ*g/mL in adiponectin, QUICKI was estimated to be higher by about 0.009 on average. All five inflammation markers were significant predictors of QUICKI. Their effects were in the hypothesized directions, with the exception of IL-6 (higher IL-6 levels were associated with higher QUICKI values, i.e., better insulin sensitivity). Furthermore, the estimated effects were not trivial. Given that the standard deviation of QUICKI was 0.03 (Table 2), the magnitude of these associations was between a quarter and a third of a standard deviation of the outcome.

### 3.3. Microvascular Structure (Capillary Density) and Function (ACR)

Table 5 summarizes the results of the multivariable analysis relating the five inflammation measures to the baseline capillary density (at rest), the maximal capillary density (after venous occlusion), and the ACR. CRP showed consistent associations with both capillary density measures (*p* = 0.009 and 0.004, resp.), while TNF*α* and PAI-1 were significantly associated only with the maximal capillary density (*p* = 0.020 and 0.009, resp.). IL-6 was also a significant predictor of both capillary density measures (*p* = 0.001 and 0.083, resp.). However, this association was not monotonic. Compared to low levels of IL-6, intermediate levels were associated with better baseline and maximal densities (on average, by 4.6 capillaries per mm^2^ and 3.9 capillaries per mm^2^, resp.). The magnitudes of these differences were about a third of the standard deviations of the two capillary density measures (8 and 12, resp., Table 2). In contrast, high levels of IL-6 were not associated with significantly different capillary densities than low levels of IL-6. None of the five inflammation measures were significantly associated with ACR.

### 3.4. Endothelial Function (FBF % Increase from Baseline to Maximal Vasodilation)

Table 6 summarizes the results of the multivariable analysis relating the five inflammation measures to endothelial function. Neither CRP nor TNF*α* were significantly associated with endothelial function (*p* = 0.721 and 0.112, resp.), but higher levels of IL-6 were monotonically associated with worse levels of FBF change from baseline to maximum vasodilation: for each additional 2 pg/mL of IL-6, there was a smaller change in FBF by 84% (*p* = 0.005). In contrast, PAI-1 and adiponectin showed associations with endothelial function in unexpected directions. Higher levels of PAI-1 were associated with stronger FBF increases (i.e., beneficial effect, *p* = 0.024), while the reverse was true for adiponectin (i.e., harmful effect, *p* = 0.088).

### 3.5. Potential Moderating Effect of BMI on the Role of the Adipokines

**Figure 1:**
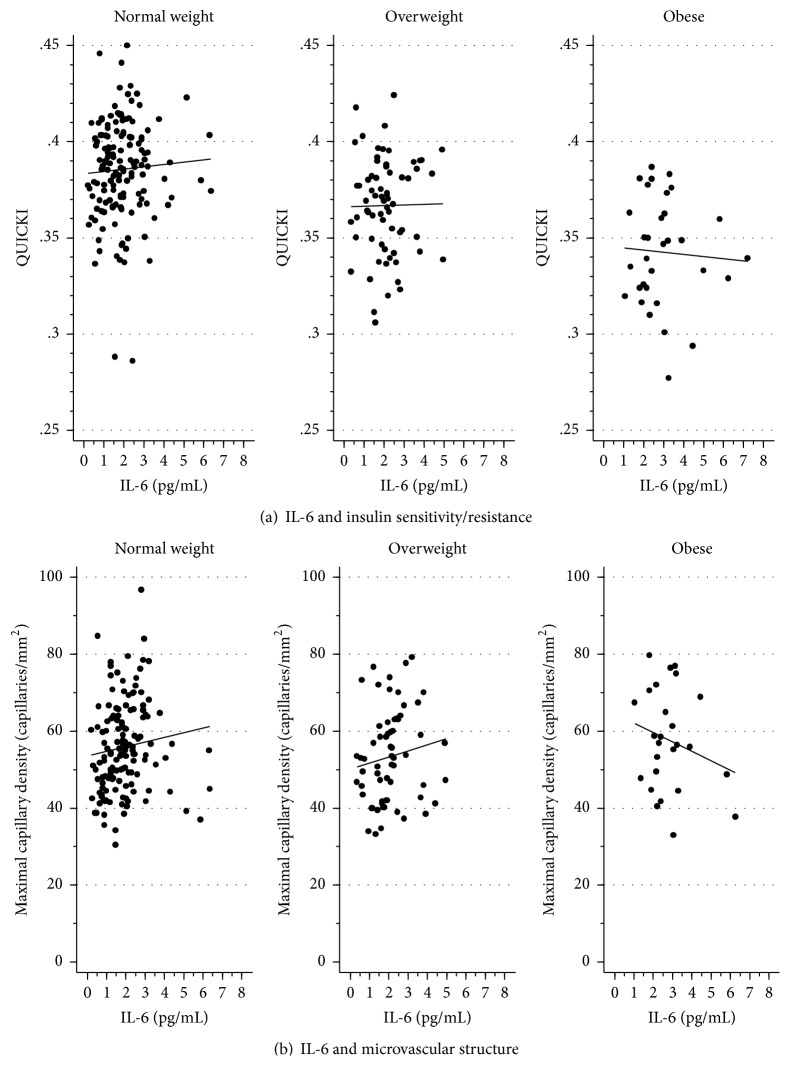
Association of IL-6 with insulin sensitivity/resistance (a) and microvascular structure (b), among normal-weight, overweight, and obese subjects.

In the main analyses, in the entire study population, IL-6 had an unexpected direction in its association with QUICKI (beneficial effect) and a nonmonotonic (inverse-U-shaped) association with capillary density (as described above, Tables 4 and 5). These patterns were apparently induced by an interaction between IL-6 and BMI (Figure 1). Among individuals of normal weight, higher IL-6 levels appeared to be associated with better outcomes (higher QUICKI and capillary densities) than lower IL-6 levels. In contrast, the reverse was true among obese individuals. However, these qualitative interactions were not statistically significant (adjusted *p* = 0.693 for QUICKI, 0.195 for baseline capillary density, and 0.835 for maximal capillary density), as the study sample was too small for such assessment. The unexpected direction of the associations of PAI-1 and adiponectin with endothelial function also appeared to be partly due to similar but nonsignificant interactions with BMI (results not shown).

### 3.6. Other Predictors of Insulin Resistance and Microvascular and Endothelial Outcomes

Regarding additional potential predictors of cardiovascular risk, African Americans had significantly lower QUICKI than Caucasians (adjusted *p* = 0.001), indicating greater insulin resistance. African Americans also had indications of lower microvascular density (*p* = 0.170 for baseline density and 0.046 for maximal density). Body mass index (BMI) had a significant independent association with both QUICKI (*p* = 0.001) and FBF % increase (*p* = 0.010), with particularly pronounced effects for obese individuals. In secondary analyses, we also found that self-reported family history of myocardial infarction or stroke was associated with worse endothelial function (*p* = 0.013 for FBF % increase).

## 4. Discussion

The majority of studies examining the association of adipokines with cardiovascular risk factors have been conducted in older and obese subjects [11]. Based on a Medline search of previously published literature, to our knowledge, this is the first study examining the association of adipokines with insulin resistance and microvascular and endothelial dysfunction in healthy young adults. Young adults without diabetes or hypertension were specifically enrolled in order to study these associations in healthy individuals, prior to clinical evidence of target organ damage. We verified the lack of target organ damage with our urine albumin excretion measurements (with only 2 of the 295 participants having had mildly elevated values).

One strength of our study is its diverse population, with sizable numbers of both African Americans and Asians. Paradoxically, African Americans are not well-studied regarding adipokines and microvascular/endothelial dysfunction, although these markers are risk factors for cardiovascular disease, which occurs more commonly and is associated with greater morbidity/mortality in African Americans compared to Caucasians [22]. Another strength of our study is that our analyses controlled for a number of participant characteristics (including BMI), in order to determine the independent associations between the inflammation markers and the outcomes of interest.

We found that higher levels of CRP, as well as of PAI-1 and TNF*α*, were independently associated with both insulin resistance and microvascular dysfunction (lower capillary density). Our CRP results are consistent with a multitude of studies showing a linear relationship between CRP and cardiovascular risk [2], including the risk of progression to diabetes mellitus [23]. CRP has also been previously correlated with both insulin resistance and endothelial dysfunction in healthy adults [24], but the average age of subjects in that study was 59 years, in contrast to 27 years in our study. PAI-1 is a key regulatory coagulation protein. Accordingly, elevated levels of PAI-1 in inflammatory and obese states are thought to contribute to the elevated cardiovascular risk via development of a prothrombotic state [2]. For TNF*α*, our findings are consistent with previous animal studies showing improved insulin sensitivity following deletion of TNF*α* or TNF*α* receptors [25] and human studies demonstrating correlation of TNF*α* with insulin resistance in the Framingham Offspring study [26]. We also found that higher levels of adiponectin were associated with higher insulin sensitivity. Adiponectin is the most highly expressed protein in adipose tissue [27] but is antiatherogenic in action, in contrast to the other adipokines [2]. Thus, our finding is consistent with the hypothesized cardioprotective effect of adiponectin.

Overall, IL-6 appeared to have an unexpected (protective and/or nonmonotonic) association with insulin sensitivity and capillary density. However, this overall finding may reflect an interaction of IL-6 with obesity; that is, higher levels of IL-6 may be associated with higher insulin sensitivity and capillary density among normal-weight individuals (beneficial effect), but an inverse (harmful) relationship may exist with these outcomes among obese individuals. In prior studies, conflicting results have been reported with regard to IL-6 in insulin resistance [28]. In vitro, IL-6 directly induces insulin resistance in adipocytes, while, in skeletal muscle cells, IL-6 appears to have an insulin-sensitizing effect [29]. Yet, circulating IL-6 levels are consistently elevated in obese and insulin-resistant individuals [28]. Therefore, it has been theorized that persistent IL-6 increases found in chronic inflammation and obesity may have detrimental effects, while transient increases in IL-6 found in lean, healthy individuals may play a physiological role in normal glucose homeostasis [28]. Supportive of this theory is the finding of beneficial effects of IL-6 in contracting muscles during exercise. Specifically in this setting, IL-6 has biological roles including induction of lipolysis and suppression of TNF production [30], in contrast to the negative effects of IL-6 in obesity and adipose tissue. Clinically, a large study of 620 women over the age of 65 overall showed that women in the highest IL-6 tertile were at higher risk of all-cause mortality [31]. However, the relative risk associated with high IL-6 was not significant among those without cardiovascular disease—healthy older women.

Our findings of a progressive change from a beneficial effect of IL-6 on both insulin sensitivity and capillary density in normal-weight individuals, to a negative effect on these outcomes in obese individuals, are intriguing and consistent with these previous reports. However, these findings are speculative, as our study was too small to formally establish the existence of an interaction between IL-6 and obesity. Furthermore, a harmful IL-6 effect on endothelial measures was uniformly present among all BMI subgroups.

None of the five inflammation measures were significantly associated with ACR in our study, although CRP [32] and PAI-1 [33] have previously been associated with microalbuminuria. As in our study, participants in those studies were normotensive, but they had somewhat higher blood pressures, underscoring the known relationship of blood pressure and age with microalbuminuria, a known cardiovascular risk factor [34]. However, subjects in these prior studies (average ages 46 and 58) were older than those in our study (average age 27). It is possible that the effect of inflammation on microalbuminuria requires a somewhat longer period of time to appear than that on other cardiovascular risk measures evaluated in this study: insulin resistance and microvascular and endothelial dysfunction.

## 5. Conclusions

Adipokine and CRP levels are significantly associated with insulin resistance and microvascular and endothelial dysfunction in young adults without diabetes or hypertension. Our study findings suggest that inflammation measures may be potential early markers of cardiovascular risk in asymptomatic individuals. Longitudinal studies of inflammation measures as early predictors of later development of cardiovascular risk factors (diabetes and hypertension) and cardiovascular events are needed.

## Figures and Tables

**Table 1:** Summary of study subject characteristics (*N* = 295, except where noted).

Age (years), mean [sd]	27 [7]
Sex, *n* (%)	
Female	187 (63)
Male	108 (37)
Race, *n* (%)	
Caucasian	160 (54)
African American	72 (24)
Asian American	63 (21)
Marital status, *n* (%)	
Not married	242 (82)
Married	53 (18)
Employment status, *n* (%)	
Student	181 (61)
Unemployed	22 (8)
Employed	92 (31)
Smoking, *n* (%)	17 (6)
Alcohol, *n* (%)	226 (77)
Weight (kg), mean [sd]	72 [17]
Body mass index (BMI, kg/m^2^),^*∗*^ mean [sd]	25.4 [5.5]
Body mass index status,^*∗*^ *n* (%)	
Normal weight (BMI < 25)	147 (58)
Overweight (BMI 25–29.9)	69 (27)
Obese (BMI 30+)	37 (15)
Systolic blood pressure (SBP, mm Hg), mean [sd]	108 [11]
Diastolic blood pressure (DBP, mm Hg), mean [sd]	66 [8]
Blood pressure status, *n* (%)	
Normotensive (SBP < 120 and DBP < 80)	249 (84)
Prehypertensive (SBP 120–139 or DBP 80–89)	46 (16)

SD: standard deviation.

^*∗*^ *N* = 252 because of missing data.

**Table 2:** Summary of inflammation measures (*N* = 295), insulin sensitivity/resistance (*N* = 293), microvascular measures (*N* = 254), urine albumin excretion (*N* = 250), and endothelial measures (*N* = 271).

Inflammation measures (*N* = 295)	
CRP (mg/L), median [iqr]	1.1 [2.0]
IL-6 (pg/mL), median [iqr]	2.0 [1.5]
TNF*α* (pg/mL), mean [sd]	9.0 [2.9]
PAI-1 (ng/mL), mean [sd]	47 [26]
Adiponectin (*μ*g/mL), median [iqr]	9.0 [7.7]

Insulin sensitivity/resistance (*N* = 293)	
Glucose (mg/dL), mean [sd]	95 [8]
Diabetes status, *n* (%)	
Normal (glucose < 100)	219 (75)
Prediabetic (glucose 100–125)	74 (25)
HOMA, median [iqr]	1.1 [0.8]
QUICKI, mean [sd]	0.37 [0.03]

Microvascular measures (*N* = 254)	
Capillary density: at rest (# capillaries per mm^2^), mean [sd]	37 [8]
Capillary density: venous occlusion (# capillaries per mm^2^), mean [sd]	55 [12]

Urine albumin excretion (*N* = 250)	
Albumin (*μ*g/mL), median [iqr]	1.9 [2.6]
Albumin (*μ*g/min), median [iqr]	1.4 [1.9]
Albumin-to-creatinine ratio (ACR, mg/g), median [iqr]	2.8 [3.8]

Endothelial measures (*N* = 271)	
FBF postischemic, mean [sd]	27 [9]
FBF ratio (postischemic to baseline), mean [sd]	9 [3]
FBF increase from baseline to postischemic (%), mean [sd]	795 [330]
FVR postischemic, median [iqr]	2.9 [1.4]
FVR ratio (baseline to postischemic), median [iqr]	0.12 [0.07]
FVR decrease from baseline to postischemic (%), mean [sd]	87 [6]

iqr: interquartile range. sd: standard deviation.

FBF: forearm blood flow. FVR: forearm vascular resistance.

**Table 3:** Correlations (*p* values) between inflammation measures and insulin sensitivity, microvascular structure and function, and endothelial function.

	QUICKI	Baseline capillary density	Maximal capillary density	ACR^**∗**^	FBF % increase
CRP^**∗**^	−0.28 (0.001)	−0.12 (0.048)	−0.13 (0.038)	−0.02 (0.802)	−0.07 (0.256)
IL-6^**∗**^	−0.11 (0.051)	0.14 (0.027)	0.16 (0.010)	0.05 (0.471)	−0.26 (0.001)
TNF*α*	−0.02 (0.752)	−0.17 (0.008)	−0.20 (0.002)	−0.05 (0.400)	0.07 (0.285)
PAI-1	−0.29 (0.001)	−0.09 (0.175)	−0.15 (0.014)	0.01 (0.932)	−0.03 (0.645)
Adiponectin^**∗**^	0.43 (0.001)	0.10 (0.125)	0.00 (0.967)	−0.02 (0.718)	0.07 (0.280)

^*∗*^These measures were log transformed because of skewness.

**Table 4:** Association of the inflammation measures with insulin sensitivity (QUICKI).

Inflammation measure		QUICKI		
	Increment	*D*	(95% CI)	*p*
CRP (mg/L)	2	−0.007	(−0.010, −0.004)	0.001
IL-6 (pg/mL)	2	0.007	(0.002, 0.011)	0.003
TNF*α* (pg/mL)	3	−0.004	(−0.008, −0.001)	0.042
PAI-1 (ng/mL)	25	−0.006	(−0.009, −0.003)	0.001
Adiponectin (*μ*g/mL)	8	0.009	(0.004, 0.014)	0.001

*D*: estimated mean difference for QUICKI, corresponding to the increment shown (~1 standard deviation) for each inflammation measure. CI: confidence interval.

The model controlled for age, sex, race, marital status, employment status, smoking, drinking, and BMI.

**Table 5:** Association of the inflammation measures with microvascular structure (capillary densities) and function (ACR).

Inflammation measure		Baseline capillary density: at rest			Maximal capillary density: after venous occlusion			ACR		
		(# capillaries per mm^2^)			(# capillaries per mm^2^)			(mg albumin per g creatinine)		
	Increment	*D*	(95% CI)	*p*	*D*	(95% CI)	*p*	*R*	(95% CI)	*p*
CRP (mg/L)	2	−1.7	(−2.9, −0.4)	0.009	−2.7	(−4.6, −0.9)	0.004	1.13	(0.96, 1.33)	0.158
IL-6 (pg/mL)	2			0.001			0.083	0.99	(0.81, 1.21)	0.918
<1.5		Ref.			Ref.					
1.5–3.4		4.6	(2.2, 6.9)	0.001	3.9	(0.5, 7.4)	0.026			
3.5+		1.2	(−2.7, 5.0)	0.554	2.4	(−3.2, 8.0)	0.397			
TNF*α* (pg/mL)	3	−1.2	(−2.5, 0.2)	0.089	−2.4	(−4.4, −0.4)	0.020	1.04	(0.87, 1.24)	0.688
PAI-1 (ng/mL)	25	−0.4	(−1.5, 0.7)	0.515	−2.2	(−3.8, −0.5)	0.009	1.12	(0.97, 1.29)	0.137
Adiponectin (*μ*g/mL)	8	0.9	(−0.8, 2.7)	0.300	−0.7	(−3.3, 1.9)	0.619	0.93	(0.74, 1.17)	0.523

ACR: albumin-to-creatinine ratio.

*D*: estimated mean difference for the outcome, corresponding to the increment shown (~1 standard deviation) for each inflammation measure. *R*: estimated geometric mean ratio for the outcome (analyzed after log-transformation because of skewness), corresponding to the increment shown (~1 standard deviation) for each inflammation measure. CI: confidence interval.

The models controlled for age, sex, race, marital status, employment status, smoking, drinking, BMI, and QUICKI.

**Table 6:** Association of the inflammation measures with endothelial function (FBF % increase from baseline to maximum vasodilation).

Inflammation measure		FBF % increase: baseline to maximum vasodilation		
	Increment	*D*	(95% CI)	*p*
CRP (mg/L)	2	9	(−39, 56)	0.721
IL-6 (pg/mL)	2	−84	(−143, −26)	0.005
TNF*α* (pg/mL)	3	42	(−10, 95)	0.112
PAI-1 (ng/mL)	25	49	(6, 91)	0.024
Adiponectin (*μ*g/mL)	8	−62	(−133, 9)	0.088

FBF: forearm blood flow.

*D*: estimated mean difference for % FBF increase, corresponding to the increment shown (~1 standard deviation) for each inflammation measure. CI: confidence interval.

The model controlled for age, sex, race, marital status, employment status, smoking, drinking, BMI, and QUICKI.

## Abbreviations

ACR: Albumin-to-creatinine ratio
BMI: Body mass index
CRP: C-reactive protein
DBP: Systolic blood pressure
FBF: Forearm blood flow
FVR: Forearm vascular resistance
IL-6: Interleukin-6
PAI-1: Plasminogen activator inhibitor-1
QUICKI: Quantitative insulin sensitivity check index
SBP: Systolic blood pressure
TNF*α*: Tumor necrosis factor alpha.
